# Acoustic Signal-Based Piezoelectric Thin-Film Microbalance: A Versatile and Portable Platform for Biomedical Sensing and Point-of-Care Testing

**DOI:** 10.3390/bios16030160

**Published:** 2026-03-13

**Authors:** Bei Zhao, Xiaomeng Li, Jing Shi, Huiling Liu

**Affiliations:** 1 School of Intelligent Manufacturing and New Energy, Xi’an Jiaotong University City College, Xi’an 710018, China; 2 The Key Laboratory of Biomedical Information Engineering of Ministry of Education, School of Life Science and Technology, Xi’an Jiaotong University, Xi’an 710049, China; 3 Bioinspired Engineering and Biomechanics Center (BEBC), Xi’an Jiaotong University, Xi’an 710049, China; 4 Engineering Research Center of Photovoltaic Technologies and Systems, Universities of Shaanxi Province, Xi’an 710049, China; 5 School of Computer and Network Engineering, Shanxi Datong University, Datong 037009, China

**Keywords:** piezoelectric microbalance, thin film, acoustic signal, deep learning, POCT

## Abstract

This study introduces a portable piezoelectric thin-film microbalance platform that combines acoustic signal analysis with deep learning for point-of-care mass detection. The system employs a flexible polyvinylidene fluoride sensor, a smartphone for acoustic signal acquisition, and three deep learning models: convolutional neural network, long short-term memory network, and Transformer. Experimental findings indicate that the Transformer achieves the highest classification accuracy of 99.5%, outperforming the convolutional neural network at 96.9% and the long short-term memory network at 97.3%, attributed to its enhanced capability to capture long-range temporal dependencies. The platform facilitates real-time, label-free detection without the necessity for bulky instrumentation, providing a cost-effective and scalable solution for decentralized diagnostics. This research establishes a foundational framework for intelligent portable micro-mass sensing with significant potential applications in precision medicine, environmental monitoring, and personalized healthcare.

## 1. Introduction

Amid the relentless advancement of modern science and technology, micro-mass sensing technology has emerged as a strategically pivotal capability across diverse disciplines, serving as a cornerstone for technological innovation and rigorous quality assurance. This necessity is especially pronounced in nanotechnology, where the precision engineering of nanomaterials and micro/nano-devices—from high-fidelity sensing elements to smart targeted delivery systems—fundamentally depends on mass characterization across the microgram (μg) to picogram (pg) scale [1,2,3,4,5]. Even minor mass fluctuations within microstructures can undermine device functionality, long-term operational stability, and biosafety profiles. Biomedical applications, in particular, demand the highest levels of measurement accuracy and reliability. Micro-mass sensing serves as a foundational tool for enabling label-free, real-time early disease diagnosis through biomarker detection, as well as for ultrasensitive quantification of biomacromolecules such as proteins, nucleic acids, and exosomes. In these life-critical contexts, measurement errors can lead to misdiagnosis, resulting in treatment failure and potential patient harm. This inherent link between measurement fidelity and clinical outcome establishes analytical precision and system robustness as fundamental requirements. Beyond its applied utility, this technology holds the potential to drive fundamental scientific discovery. Confronted with global trends toward extreme miniaturization and escalating system complexity, conventional mass detection methodologies exhibit inherent limitations in sensitivity, spatial resolution, and dynamic response. The development of next-generation micro-mass sensing platforms not only optimizes manufacturing workflows and enhances resource efficiency but also establishes indispensable experimental infrastructure for frontier research in surface science, molecular interaction kinetics, single-molecule biophysics, and real-time biomolecular binding assays. Consequently, deepening fundamental investigations and broadening the application scope of micro-mass sensing technology carry profound scientific implications and urgent strategic relevance, positioning it as a critical enabler for the future advancement of precision biomedicine, diagnostics, and next-generation advanced manufacturing [6,7,8].

The rapid and high-precision detection of ultra-trace analytes represents a pivotal scientific challenge driving innovation across interdisciplinary domains. While high-energy colliders in particle physics enable subatomic-scale particle detection with exceptional spatial and energy resolution, their dependence on massive accelerator infrastructure incurs prohibitive capital and operational costs alongside substantial energy demands, limiting accessibility to specialized facilities. Although atomic force microscopy (AFM) delivers atomic-resolution topographical and nanomechanical characterization, its serial point-scanning mechanism fundamentally constrains analytical throughput and precludes efficient large-area sampling, while also imposing stringent environmental requirements such as vibration isolation and thermal stability. Surface plasmon resonance (SPR) enables real-time, label-free monitoring of biomolecular interactions, yet remains inherently susceptible to nonspecific binding, temperature fluctuations, and refractive index variations within the sensing medium. Data interpretation further necessitates complex optical modeling and curve-fitting procedures, which can undermine measurement reproducibility and constrain broader adoption. Collectively, these established methodologies confront persistent limitations—including exorbitant instrumentation costs, narrow dynamic ranges, low throughput, environmental sensitivity, and cumbersome data interpretation workflows—which not only constrain fundamental scientific inquiry but also impede scalable deployment in critical applications such as clinical diagnostics, environmental monitoring, and industrial quality control. Consequently, there exists an urgent imperative to develop next-generation ultra-trace detection platforms that synergistically integrate femtogram-level mass sensitivity, wide dynamic range, cost-effective manufacturability, operational simplicity, high-throughput capability, environmental robustness, and standardization potential. The realization of such advanced sensing paradigms promises to catalyze transformative advances in precision medicine, point-of-care testing (POCT), single-molecule biophysics, and intelligent diagnostic systems, ultimately fostering a new era of accessible, reliable, and versatile analytical science [9,10,11,12,13,14,15,16].

In the domain of micro-mass detection, piezoelectric sensing has emerged as a highly promising paradigm, distinguished by its exceptional sensitivity, real-time responsiveness, and operational simplicity. This approach exploits the intrinsic electromechanical coupling properties of piezoelectric materials: under alternating electric field excitation, piezoelectric resonators—such as Quartz Crystal Microbalance (QCM), Thin-Film Bulk Acoustic Resonator (FBAR), and Surface Acoustic Wave (SAW) devices—generate stable mechanical oscillations via the inverse piezoelectric effect [17,18,19,20,21]. Upon specific binding of target analytes (e.g., nanoparticles, proteins, nucleic acids, or exosomes) to a functionalized sensing interface, the added mass incrementally increases the effective mass of the vibrational system, inducing a quantifiable shift in resonant frequency. Under rigid, uniform, and thin-film loading conditions, this frequency shift rigorously adheres to the Sauerbrey equation, exhibiting a linear negative correlation proportional to the adsorbed mass, thereby establishing a direct, calibration-based quantitative model that circumvents complex signal transduction. Coupled with high-precision frequency measurement systems and pre-calibrated reference curves, the methodology enables millisecond-scale dynamic tracking and accurate quantification of mass [22,23,24,25,26,27,28,29,30,31,32,33,34,35]. Compared to alternative sensing modalities, piezoelectric sensing offers compelling advantages—including ultrafast response (millisecond-scale), minimal power consumption (microwatt-level), broad dynamic range, superior environmental robustness, and inherent compatibility with miniaturization and system integration. These attributes confer exceptional versatility across diverse applications: real-time online monitoring of airborne nanoparticles in environmental science; in situ mass monitoring during thin-film deposition and etching processes in microelectronics manufacturing; and label-free, real-time analysis of biomolecular interactions (e.g., antigen–antibody binding kinetics, DNA hybridization, cellular adhesion dynamics) in biomedicine, serving as a critical enabler for POCT, disease biomarker screening, and drug development. Functioning as a pivotal bridge between fundamental transduction principles and practical implementation, piezoelectric sensing not only significantly enhances the efficiency and reliability of trace-mass detection but also lays a robust foundation for advancing precision detection science toward integrated, intelligent, and field-deployable platforms. By empowering researchers to detect trace analytes with unprecedented speed and accuracy, this technology catalyzes interdisciplinary innovation and unlocks transformative possibilities across scientific and technological frontiers [36].

Recent progress in miniaturized mass sensing has shifted focus toward device downsizing and portability-driven design, enabling high-precision micro-mass measurement beyond traditional laboratory settings. These engineered systems now support deployment in field applications such as on-site environmental monitoring, point-of-care testing, and distributed sensor networks. This evolution encompasses not merely physical hardware reduction but also systematic innovation in signal output architectures and human–machine interaction paradigms, fostering an intelligent, intuitive, and field-deployable detection experience. Specifically, the system replaces traditional wired interfaces and bulky display units with acoustic signals as the medium for result transmission. This design paradigm substantially reduces device footprint and energy consumption—critical enablers for miniaturization—while ensuring robust wireless data transfer suitable for dynamic field environments [37,38,39,40]. Moreover, deep integration with mobile intelligent terminals (e.g., smartphones and tablets) establishes a lightweight, interactive data visualization platform: users can monitor results in real time, dynamically adjust experimental parameters, and perform data storage, annotation, and cloud synchronization through an intuitive graphical user interface (GUI). The synergistic fusion of communication and mobile ecosystem infrastructure culminates in a highly integrated, low-power, and intelligent micro-detection platform. This paradigm effectively transcends spatial and infrastructural limitations inherent to laboratory settings, providing a viable technical solution for precise mass analysis in field operations, bedside diagnostics, and resource-constrained contexts. Simultaneously, it establishes a robust foundation for next-generation Internet of Things (IoT)-enabled distributed sensing architectures, heralding a transformative shift toward accessible, adaptive, and interconnected analytical technologies [41,42,43,44].

Under alternating electric field excitation, piezoelectric thin-film resonators undergo high-frequency mechanical vibration through the inverse piezoelectric effect, concurrently emitting acoustic signals that capture the dynamic characteristics of the system. Spectral parameters derived from these signals, including shifts in resonant frequency, variations in quality factor, and phase response, correlate directly with minute mass loads deposited on the sensor surface. This physical relationship provides the basis for a noninvasive, label-free approach to micro-mass detection. Nevertheless, practical implementation encounters significant challenges: acoustic signals possess inherently low amplitude, are highly susceptible to environmental noise and mechanical vibrations, and the mass–signal mapping relationship frequently manifests nonlinear and multivariable coupling behaviors, rendering conventional signal processing techniques inadequate for robust feature extraction and precise quantitative modeling. Recent breakthroughs in artificial intelligence (AI), particularly deep learning (DL) methodologies for time-series signal analysis, offer transformative solutions. Architectures such as Convolutional Neural Networks (CNNs) and Long Short-Term Memory (LSTM) networks enable adaptive learning of hierarchical feature representations from acoustic waveforms, effectively suppress noise interference, and construct high-precision, generalizable mass–acoustic response mapping models. Embedded AI systems leveraging lightweight neural networks have already demonstrated end-to-end acquisition, processing, and visualization of acoustic signals, exhibiting notable advantages in low power consumption, real-time operation, and field-deployability across applications such as environmental particulate monitoring and industrial process control. However, the potential of this integrated strategy for ultra-trace detection remains underexplored and insufficiently validated. Accordingly, researchers are actively advancing the synergistic integration of acoustic signal analytics with Piezoelectric Thin-Film Microbalance (PTFM) technology through AI-enhanced acoustic feature decoding frameworks that efficiently transduce resonator-emitted acoustic waves into high-sensitivity mass-sensing data. Preliminary investigations confirm that this approach substantially enhances detection efficiency and quantitative accuracy while minimizing reliance on complex peripheral circuitry. This convergent methodology not only expands the theoretical and methodological frontiers of piezoelectric sensing but also furnishes critical technical foundations for next-generation intelligent micro-mass sensing systems targeting POCT, single-molecule analysis, and IoT node deployment—marking a pivotal milestone in the evolution of micro-nano sensing toward intelligence, miniaturization, and universal applicability [45,46].

Building upon the aforementioned technical foundations and research advancements, this study systematically advances the innovation and translational development of portable piezoelectric micro-mass sensing technology through two pivotal objectives. First, we designed and fabricated a high-sensitivity PTFM by optimizing resonator geometry and surface biofunctionalization strategies to achieve a stable and reproducible detection of mass variations at the microgram (μg) level. Second, we developed AI algorithms specifically engineered for acoustic signal interpretation to construct a highly robust, nonlinear mapping model between acoustic features and mass loading, thereby substantially enhancing signal decoding accuracy, noise resilience, and cross-scenario generalization capability. The translational impact is profound: accelerating early infectious disease screening and real-time surveillance; empowering POCT in resource-limited settings (e.g., primary care clinics, field deployments, and remote communities) to strengthen public health responsiveness and equity; and providing a reliable analytical platform for personalized therapeutic monitoring, chronic disease management, and precision medicine implementation.

While piezoelectric microbalances have traditionally relied on electrical impedance analysis for mass detection, our work pioneers a paradigm shift toward acoustic signal transduction and intelligent interpretation. Unlike conventional QCM and Thin-FBAR systems that require sophisticated frequency counting circuitry and controlled laboratory environments, our platform leverages the inherent acoustic signals from piezoelectric vibration as the primary sensing modality. This approach fundamentally reconfigures the sensor architecture, eliminating the need for direct electrical contact with the resonator during measurement—a critical advantage for biofluid applications where electrical isolation is essential. Furthermore, while previous smartphone-assisted sensing platforms have primarily focused on optical or basic electrical measurements, our integration of DL-based acoustic analysis represents a significant advancement in extracting high-fidelity information from complex acoustic signals captured by commodity hardware.

## 2. Principle Analysis

### 2.1. Piezoelectric Effect and Piezoelectric Microbalance

The piezoelectric effect denotes a fundamental physical phenomenon exhibited by materials possessing non-centrosymmetric crystalline structures—such as quartz, barium titanate, and lead zirconate titanate (PZT)—which facilitates reversible energy transduction between mechanical and electrical domains. This phenomenon is categorically differentiated into the direct piezoelectric effect (occasionally referenced as the positive piezoelectric effect in specific literature) and the converse piezoelectric effect, contingent upon the direction of energy conversion. In the direct piezoelectric effect, the imposition of external mechanical stress induces microscopic lattice distortion within the crystal framework, resulting in the relative displacement of positive and negative charge centers. This displacement consequently generates a surface-bound charge distribution and a measurable electric potential difference linearly proportional to the applied stress. Conversely, under the converse piezoelectric effect, an externally applied electric field triggers the oriented rearrangement of intrinsic electric dipole moments or lattice strain, thereby eliciting a macroscopic mechanical deformation commensurate with the field intensity. Collectively, these reciprocal mechanisms constitute the foundational physical principle underpinning the functionality of piezoelectric materials in advanced engineering applications, including high-precision sensors, micro-displacement actuators, ultrasonic transducers, and vibration energy harvesters. Their pivotal role underscores substantial theoretical significance and broad applicability across interdisciplinary fields such as smart materials science and micro/nano-electromechanical systems (MEMS/NEMS).

The operational principle of the PTFM is fundamentally grounded in the converse piezoelectric effect and the quantitative frequency–mass relationship governing resonant microsystems. The core sensing element comprises a microelectromechanical resonator integrating a piezoelectric thin film (e.g., AlN, ZnO, or PZT) sandwiched between patterned electrodes. Upon application of an alternating voltage, the film undergoes cyclic mechanical deformation via the converse piezoelectric effect, exciting a stable thickness-shear mode resonance at a characteristic fundamental frequency $f_{0}$, which is intrinsically determined by the film’s material properties, geometric dimensions, boundary conditions, and elastic constants.

When the target analytes adsorb onto the functionalized active surface, the incremental areal mass loading Δm perturbs the resonant system. Under the assumptions of rigid, thin, and uniformly distributed mass loading—and operation in the linear elastic regime—the resonant frequency shift Δf is quantitatively described by the Sauerbrey equation:

(1) $$∆f=\;-\frac{2f_{0}^{2}}{A\sqrt{\rho_{q}\mu_{q}}}∆m$$

where Δf denotes the resonant frequency shift (Hz), $f_{0}$ is the unloaded fundamental resonant frequency (Hz), A is the piezoelectrically active area (cm^2^), $\rho_{q}$ and $\mu_{q}$ represent the density (g·cm^−3^) and shear modulus (g/(cm·s^2^)) of the resonator substrate, and Δm is the areal mass change (g·cm^−3^). The negative sign signifies an inverse proportionality: increased mass loading induces a measurable decrease in resonant frequency. This linear relationship establishes a rigorous basis for gravimetric quantification. High-precision frequency tracking systems monitor Δf in real time; combined with a pre-calibrated conversion coefficient derived from Equation (1), the adsorbed mass Δm is inversely calculated with sub-nanogram resolution.

Mass sensitivity $S_{M}$, defined as the magnitude of frequency response per unit mass change, serves as a key performance metric. The theoretical sensitivity derived from Equation (1) can be expressed as follows:

(2) $$S_{M}=\left|\frac{\Delta f}{\Delta m}\right|=\frac{2f_{0}^{2}}{A\sqrt{\rho_{q}\mu_{q}}}$$

indicating that sensitivity scales quadratically with $f_{0}$ and inversely with active area and substrate acoustic impedance. However, practical implementations are subject to deviations arising from residual stress during thin-film deposition, viscoelastic effects of soft adsorbates, temperature drift, and non-ideal boundary conditions.

Consequently, experimental calibration is essential, yielding the empirically determined sensitivity:

(3) $$S_{m}=-\frac{\Delta f}{\Delta m}$$

where Δf and Δm are the values measured under controlled conditions. Maximizing $S_{m}$ while ensuring stability and repeatability remain a central objective in PTFM design.

To achieve high-fidelity detection across diverse applications—such as trace gas sensing, biomolecular binding assays, or environmental particulate monitoring—the resonator architecture must be co-optimized with surface chemistry (e.g., receptor functionalization for selective adsorption), environmental compensation strategies, and signal processing protocols. Rigorous minimization of non-specific binding, damping losses, and external interferences further enhances reliability. Through this integrated approach, PTFMs deliver exceptional mass resolution, establishing their critical role in advanced sensing platforms within MEMS/NEMS, point-of-care diagnostics, and real-time environmental analytics.

### 2.2. PVDF Piezoelectric Microbalance

Polyvinylidene fluoride (PVDF) is a semi-crystalline fluoropolymer that exhibits pronounced piezoelectric activity following uniaxial stretching and high-voltage poling processes. This treatment facilitates an efficient transition of polymer chains from the non-polar α-phase conformation to the highly polar *β*-phase with strong dipole orientation, thereby enabling stable macroscopic piezoelectric responses. PVDF films are characterized by their exceptional mechanical flexibility, low acoustic impedance compatible with biological tissues, excellent chemical stability, and biocompatibility. While the piezoelectric response intensity and electromechanical energy conversion efficiency of PVDF are moderate compared to traditional rigid inorganic piezoelectric materials such as quartz or PZT, its advantages lie in solution processability, lightweight structure, mechanical robustness against impacts, and environmental adaptability, establishing an irreplaceable position in flexible and wearable sensing applications [47,48].

In the context of PTFM technology, PVDF films can be configured as mass-sensitive resonant elements operating in thickness-extensional vibration mode. When minute masses were adsorbed onto the sensing surface, the effective vibrating mass increased, leading to a reversible decrease in resonant frequency. Under ideal conditions where the added mass is small, the adhered layer is uniform and rigid, there exists a clear negative linear correlation between the frequency shift and the mass loading on the surface. Under ideal conditions wherein the adsorbed areal mass loading $\Delta m_{A}$ is substantially smaller than the areal mass density of the resonator (ρt), and the adsorbed layer is rigid and uniformly distributed, the frequency shift Δf exhibits a linear dependence on $\Delta m_{A}$, described by the following relationship:

(4) $$\Delta f=-\frac{f_{0}}{2\rho t}\Delta m_{A}$$

where $f_{0}$ denotes the fundamental unloaded resonant frequency, ρ represents the material density, and t signifies the film thickness. This theoretical model elucidates that the mass sensitivity scales directly with the resonant frequency $f_{0}$, while exhibiting an inverse dependence on both the material density ρ and the film thickness t.

The detection sensitivity is influenced by the fundamental resonant frequency, intrinsic material properties, and geometric dimensions of the film. It should be noted that the quality factor of PVDF resonant structures is typically lower than that of highly rigid crystal-based microbalances, which limits their ultimate resolution and environmental noise rejection capabilities. However, the inherent flexibility of PVDF endows the devices with superior conformability to curved surfaces, potential for wearable integration, and feasibility for large-area array fabrication. These features make PVDF particularly valuable in scenarios where conventional rigid sensors are unsuitable, such as complex curved surface monitoring, flexible electronic skins, and implantable bio-interfaces.

### 2.3. AI Algorithms Basis

As a forefront interdisciplinary field, AI aims to simulate, extend, and enhance human cognitive capabilities. In recent years, AI has demonstrated significant application value and developmental potential in critical sectors such as industrial manufacturing and healthcare. Machine Learning (ML), one of the core components of AI, enables systems to autonomously uncover latent patterns and structural relationships within data through a data-driven mechanism, thereby accomplishing tasks like prediction, classification, and clustering without reliance on predefined explicit rules. With advancements in computational hardware performance and the maturation of big data technologies, DL, an advanced ML approach based on deep neural network architectures, has further propelled this progress. Architectures such as CNNs, Recurrent Neural Networks (RNNs), and Transformers can perform end-to-end hierarchical feature extraction directly from raw sensor signals (such as vibration and acoustic data), automatically generating highly discriminative representations that provide robust support for subsequent classification and regression tasks. Against this backdrop, the application of AI technologies, particularly deep learning methods, to the domain of piezoelectric vibration acoustic signal recognition not only facilitates the development of intelligent adaptive microbalance systems but also serves as a pivotal driving force for advancements in POCT and wearable sensing technologies. This integration underscores the vast potential of AI in the realms of precision sensing and intelligent diagnostics, especially highlighting its immense application prospects in enhancing detection accuracy and the level of intelligence.

In ML, neural network-based models, particularly those within DL, have become essential for addressing complex tasks. Two pivotal models in this context are CNNs and RNNs. A CNN is a specialized neural network model inspired by the human visual nervous system. As a multilayer perceptron, it incorporates ‘local sensing’ and ‘weight sharing’ features. Local sensing targets specific regions of the input data, whereas weight sharing reuses parameters to reduce computational complexity. These features render CNNs efficient on devices with limited computational resources. CNNs have demonstrated efficacy in tasks involving visual and sequential data and have been successfully applied to image classification, object recognition, and detection. They remain the predominant approach for image identification and speech recognition tasks. The RNN is characterized by the addition of feedback connections to the traditional feedforward structure. Conventional feedforward networks process data sequentially without feedback loops, limiting their capacity to handle sequential data. RNNs address this limitation by incorporating feedback connections, enabling them to retain the memory of past input data. However, traditional RNNs encounter challenges with vanishing or exploding gradients during training, which hinders the model’s ability to learn long-term dependencies. LSTM networks resolve this issue by employing memory blocks with gating mechanisms instead of conventional hidden neurons. These gates regulate the information flow, mitigate gradient issues, and allow LSTMs to learn long-term dependencies in sequential data, thereby outperforming traditional RNNs in tasks such as machine translation and speech recognition.

The Transformer, a deep neural network architecture grounded in self-attention mechanisms and originally devised for natural language processing, models sequence dependencies by computing position-wise attention weights to dynamically aggregate contextual information without recurrence. Its core framework employs query-key-value operations, positional encoding for temporal coherence, and multi-head attention to capture heterogeneous feature representations. Increasingly adopted in signal analysis—particularly for vibration and acoustic time-series data—Transformers effectively discern long-range temporal dependencies and cross-channel correlations in multi-sensor systems, enabling robust end-to-end classification. Targeted adaptations have emerged, including hybrid CNN-Transformer architectures for localized feature extraction, computationally efficient attention variants (e.g., sparse or local attention), and self-supervised pre-training to improve data efficiency. While less mature than established CNNs and RNNs, Transformers demonstrate compelling potential for intelligent analysis of high-dimensional, multi-source time-series under complex operational conditions. However, their “black-box” interpretability limitations and hyperparameter sensitivity (e.g., attention head count, positional encoding scheme) may challenge practitioner trust relative to more transparent and empirically validated architectures.

## 3. Materials and Methods

### 3.1. Experimental Setup

**Figure 1:**
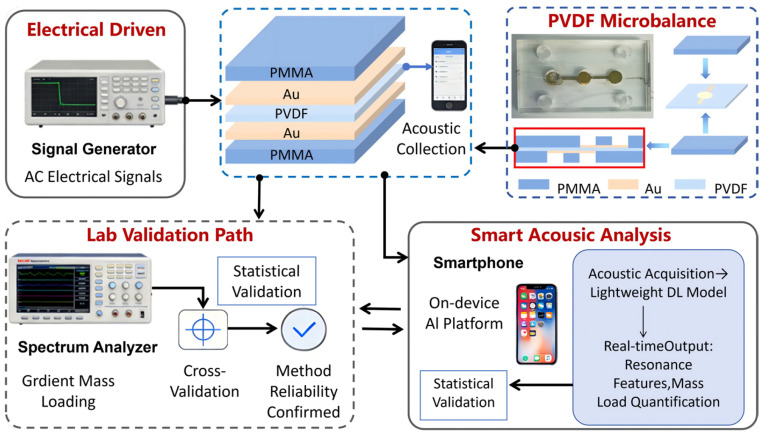
System architecture of the portable acoustic sensing platform for POCT applications. Solid lines denote the field-deployable workflow; dashed lines indicate the laboratory validation pathway.

In this study, A portable acoustic sensing experimental system tailored for POCT applications was developed. The system comprises a PTFM sensor, a programmable electrical signal excitation source, a smartphone-based acoustic signal acquisition module, and an intelligent analysis platform integrated with different lightweight DL algorithms. The overall system architecture is illustrated in Figure 1.

The piezoelectric thin-film microbalance sensor features a sandwich-structured diaphragm configuration consisting of two patterned gold (Au) electrode layers, a PVDF piezoelectric active layer, and a polymethyl methacrylate (PMMA) supporting substrate layer. The PVDF functional layer (thickness: 28 μm) is a flexible, lead-free piezoelectric polymer that exhibits superior electromechanical coupling properties and mechanical compliance. The PMMA substrate provides structural rigidity and precisely enforces the clamped boundary conditions along the diaphragm perimeter. High-purity gold electrodes, symmetrically patterned on both sides of the PVDF film via DC sputtering, ensured low-loss transmission of piezoelectrically induced signals. The device fabrication involved micropatterning of the PMMA substrate, deposition of patterned electrodes onto the PVDF surface, and precise alignment and lamination of the upper and lower support layers using a high-adhesion optically clear adhesive (OCA), yielding a flexible sensing unit with robust structural integrity and excellent interfacial adhesion.

The electrical signal generator, functioning as a broadband excitation source, generates standard waveforms (sine, square, and triangular) with a frequency range of several hertz to several megahertz and an amplitude adjustment range of millivolts to volts. To satisfy the portability and accessibility requirements inherent to POCT deployment, a commercial smartphone sourced from Xiaomi Technology Co., LTD (Mi 11 Ultra, Beijing, China, with built-in microphone sampling rate: 48 kHz) was employed as the acoustic acquisition terminal to capture the acoustic emissions generated by the sensor vibration in real time. The raw acoustic data were transmitted to the processing platform via Bluetooth or a USB interface. The analytical core incorporates a lightweight DL algorithm optimized for efficient on-device deployment on mobile terminals, enabling real-time edge-side processing, including time–frequency feature extraction, resonance mode identification, and quantitative inversion of the loaded mass. This architecture substantially reduces the dependency on specialized laboratory instrumentation, thereby enhancing the feasibility of rapid field-deployable diagnostics.

To validate the accuracy and robustness of the DL-based analytical outputs, a high-precision spectrum analyzer was used to independently measure the resonance spectra of the sensor under incrementally increased mass-loading conditions.

### 3.2. Experimental Materials

This study presents the development of a microbalance-sensing device based on a piezoelectric thin film. The primary sensing component is an ultra-thin, lead-free PVDF thin film characterized by a high *β*-phase crystalline content. This material exhibits exceptional piezoelectric responsiveness and mechanical flexibility, along with integrated benefits such as a broad frequency response, high electromechanical conversion sensitivity, structural robustness, low density, and excellent processability. Owing to its efficient bidirectional electromechanical coupling capability, which facilitates the reversible conversion between mechanical and electrical energy, PVDF has become a crucial functional material in the advancement of piezoelectric biomedical micro/nano-devices. During the assembly of the device, a 28 μm-thick PVDF thin film (M.S.I., Westborough, MA, USA) was utilized as the active sensing layer; a 1 mm-thick PMMA plate (sourced from Shenzhen, China) served as the rigid substrate; interlayer bonding was achieved using a 50 μm-thick double-sided pressure-sensitive adhesive film (3M Company, St. Paul, MN, USA); and high-purity gold thin films (KJ Group, Hefei, China) were deposited to form the top and bottom electrode layers to ensure optimal electrical conductivity and interfacial stability. This meticulously engineered material architecture and structural configuration collectively provide a robust foundation for the high-sensitivity mass detection performance of the microbalance.

### 3.3. Experimental Procedures

The experimental procedures are outlined as follows:

First, the signal generator was configured to produce an excitation electrical signal with predefined parameters. Critical parameters, including frequency, amplitude, and waveform, were precisely regulated in accordance with the experimental design requirements to ensure that the excitation signal frequency approximated the resonant frequency of the PTFM sensor.

Subsequently, an excitation electrical signal was delivered to the PTFM sensor. The excitation electrical signal, functioning as an external mechanical excitation applied to the piezoelectric-sensitive layer, induced mechanical vibration via the inverse piezoelectric effect. Acoustic signals were generated during vibration and were directly acquired using a smartphone. The smartphone utilized in the experiment was a Mi 11 Ultra. During data acquisition, the microphone of the smartphone was positioned on the same horizontal plane as the microbalance sensor, at a distance of approximately 20 cm. Both the smartphone and the microbalance sensor were placed inside an enclosure constructed from soundproof cotton, and the entire experiment was conducted in a quiet laboratory setting.

**Figure 2:**
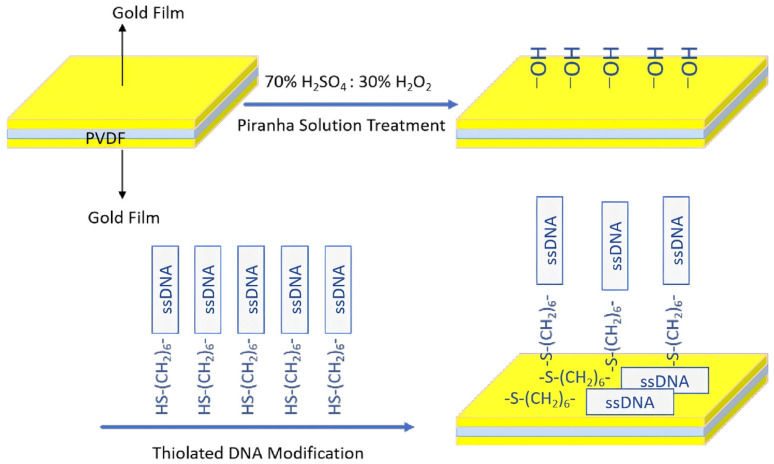
Process of nucleic acid immobilization.

During the data processing phase, spectral analysis was performed on the acquired data, with both the raw acoustic waveforms and their corresponding frequency-domain representations serving as inputs. Subsequently, CNN, LSTM, and transformer models were employed to analyze the signals, culminating in classification. In the mass detection experiment, following the sensor calibration and determination of the baseline resonant frequency, a quantified volume of the analyte (e.g., single-stranded DNA) was deposited onto the sensing surface, as illustrated in Figure 2.

As the target analytes were adsorbed and accumulated at the interface, the effective mass of the sensor increased, inducing a measurable shift in the resonant frequency of the piezoelectric thin film. The mass of the adsorbed substance was quantified based on the established correlation between the acoustic signals and the adsorbed mass. Simultaneously, the frequency–time profile was monitored in real time using a spectrum analyzer, and the mass variation in the adsorbed layer was quantitatively inverted according to the Sauerbrey equation. This integrated methodology achieved precise mass detection and enabled a systematic comparison of the aforementioned results. All the experimental procedures were carried out in a quiet laboratory.

### 3.4. AI Algorithms

Three distinct DL algorithms that are readily deployable on smartphone terminals were employed to analyze the acoustic signals. For the DL algorithms, 2500 raw data samples of each mass load type were utilized, amounting to a total of 25,000. Each sample represents a 0.1 s segment of the acoustic signals. At a sampling rate of 48 kHz, this yields exactly 4800 individual data points per data sample. All samples were acquired as statistically independent measurements with rigorous procedural safeguards against bias or contamination and no sample shares temporal, procedural, or environmental context with another. Independence was ensured through: (i) separate experimental sessions conducted on non-consecutive days; (ii) randomized mass-load sequence per session; (iii) environmental monitoring (25 ± 1 °C, 50 ± 5% RH). Of these, 60% were allocated for network training, 20% for validation during training, and the remaining 20% for testing the accuracy of the network.

CNNs utilize fundamental mechanisms, such as local perception, weight sharing, and hierarchical feature extraction, to effectively extract essential information from raw data. In the realm of acoustic signal detection, CNN architectures typically commence with an input layer that receives one-dimensional acoustic signals or, more frequently, their two-dimensional representations, such as time-frequency spectrograms produced via Short-Time Fourier Transform (STFT) or Wavelet Transform (WT). Subsequently, the convolutional layers employ multiple learnable kernels to scan the input and extract local features, such as energy variations within specific frequency bands and local signal patterns. Activation functions such as ReLU introduce nonlinearity into the network, enabling the capture of complex nonlinear relationships. Pooling layers, such as max pooling, down-sample these feature maps, thereby reducing the dimensionality and computational complexity while preserving key features and enhancing robustness against minor perturbations. This hierarchical process allows shallow layers to capture basic patterns, whereas deeper layers progressively develop a sophisticated understanding of the acoustic features. Features extracted through multiple convolutional and pooling layers are then flattened and fed into fully connected layers for integration before being passed to the output layer, which often employs a Softmax function to provide probabilities of samples belonging to different quality load states.

**Figure 3:**
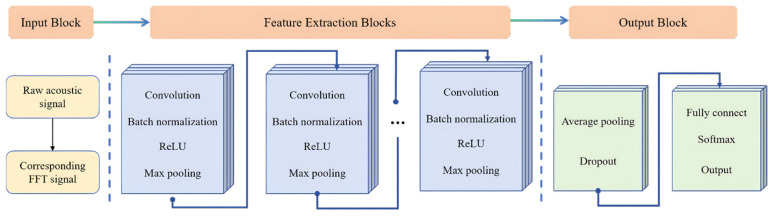
CNN structure diagram. The input block, the feature extraction blocks and the output block were stacked.

In this study, a DL algorithm architecture based on a CNN was developed, beginning with an input layer designed to receive raw acoustic signals along with their corresponding FFT signals. Subsequently, combination feature extraction blocks incorporating convolutional, batch normalization, ReLU, and max pooling layers were employed for feature extraction. An average pooling layer and a dropout layer were subsequently introduced to mitigate overfitting. The final output was achieved using a combination of fully connected, Softmax, and output layers. A schematic illustration of the CNN-based deep-learning architecture is shown in Figure 3.

**Figure 4:**
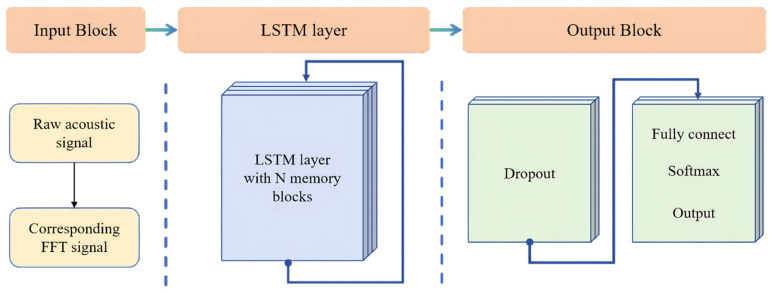
RNN structure diagram. The input layer, the LSTM layer and the output block were stacked.

RNNs are adept at processing sequential data owing to their internal memory, which allows them to maintain temporal dependencies by backpropagating hidden states across time steps. Consequently, they are particularly well-suited for tasks involving time-series data. However, standard RNNs face challenges, such as the vanishing or exploding gradient problem, when learning long-term dependencies. LSTM networks address this issue through a specialized architecture comprising a cell state and three interacting gates: the forget gate, which determines which information to discard from the cell state; the input gate, which regulates the incorporation of new information; and the output gate, which decides which portion of the cell state to output. This configuration enables LSTM networks to learn and retain pertinent information in lengthy sequences effectively. LSTM networks are particularly valuable for directly analyzing time-varying or acoustic signals in sequence form, as they can capture the dynamic evolution and temporal correlations of signal patterns, thereby accurately classifying acoustic signals of varying load qualities. A schematic illustration of the LSTM-based DL architecture is shown in Figure 4. The DL algorithm architecture based on RNN begins with a sequence input layer for inputting the original vibration signal, akin to the CNN model and the corresponding FFT signal. LSTM layers with varying numbers of memory blocks (ranging from 20 to 160) were employed to extract features, and a dropout layer with a 20% dropout rate was utilized to prevent overfitting. Finally, a combination of fully connected, softmax, and output layers was used to produce the classification results. The Adam optimizer was employed to train the RNN deep network.

**Figure 5:**
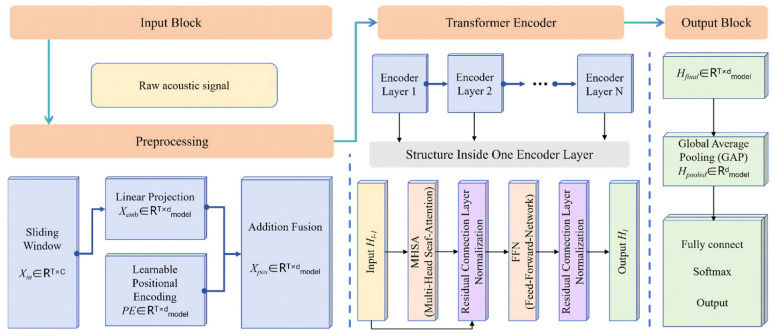
Transformer structure diagram. The input block, the transformer encoder and the output block were stacked.

A Transformer-based model specifically tailored for the task of acoustic signal classification under varying mass loads was proposed. Initially, the original one-dimensional time series signals were subjected to preprocessing, utilizing the sliding window technique to segment them into fixed-length patches $X_{in}\in R^{T\times C}$, where T denotes the block length and C represents the number of signal channels (in this study, *c* = 1). These patches are subsequently mapped to the designated feature dimension dmodel via a linear projection layer, resulting in an initial embedding $X_{emb}$. To retain temporal sequence information, a learnable positional encoding (*PE*) was incorporated, yielding a position-aware input sequence $X_{pos}=X_{emb}+PE$. The core of the model comprises L stacked transformer encoder layers. Each encoder layer consists of two principal components: multi-head self-attention (MHSA) and a position-aware feedforward network (FFN), both followed by residual connections and layer normalization. The MHSA captures long-range temporal dependencies within the signal, whereas the FFN augments the representational capacity of the model through nonlinear transformation. Following processing by the L-layer encoder, the final sequence representation, *H_final_* was obtained. A global average pooling (GAP) operation is then applied to sequence dimension T to produce a fixed-size feature vector *H_pooled_*. This vector was subsequently processed through one fully connected layer and passed through a softmax activation function, yielding a 10-dimensional probability distribution that indicated the confidence score for each mass load category, thereby completing the classification. A schematic illustration of the Transformer -based DL architecture is shown in Figure 5.

## 4. Results and Discussion

### 4.1. Data Presentation

**Figure 6:**
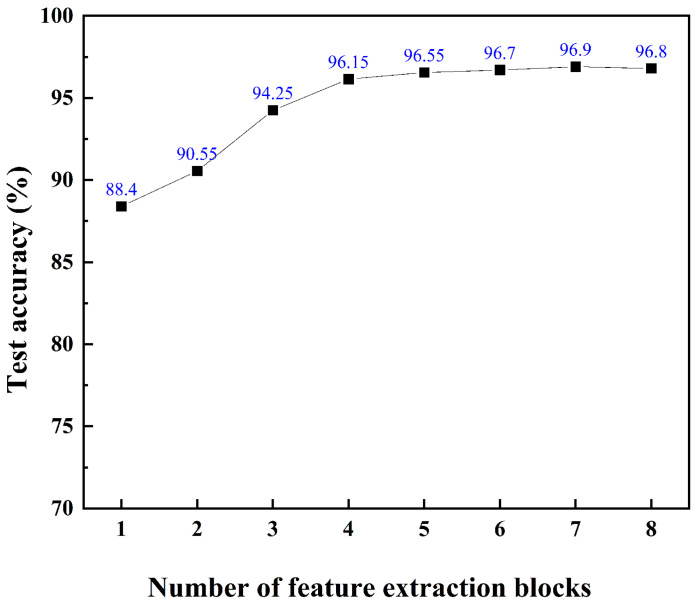
Relationship between test accuracy and the number of feature extraction blocks. The seven-layered CNN achieved the highest test accuracy.

**Figure 7:**
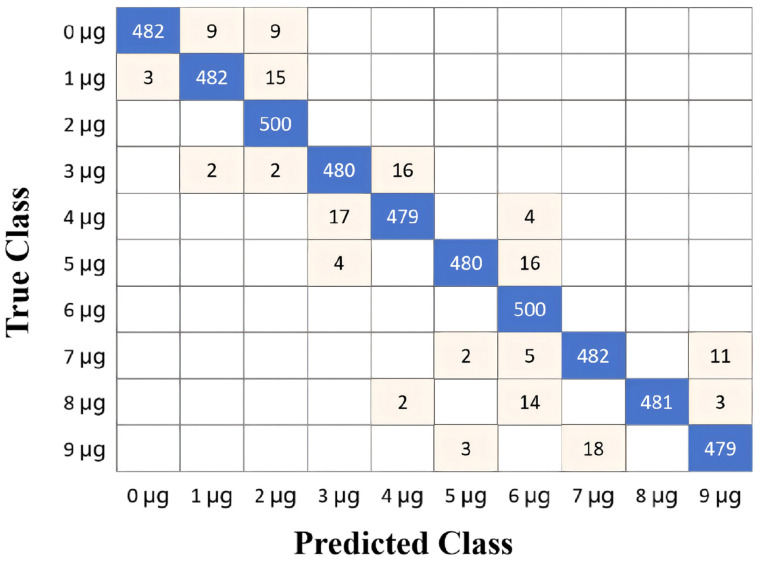
Confusion matrix for test data showing the classification results using the CNN model with seven feature extraction blocks. (*n* = 5000 samples; 500 per class: 0 to 9 μg; Overall accuracy: 96.9%).

Figure 6 shows the mean accuracy of the CNN test results across various configurations of the combined feature extraction blocks. The CNN models achieved a test accuracy exceeding 95% when the number of feature extraction blocks was greater than four, with the highest test accuracy of 96.9% at seven feature extraction blocks. Each step of DL was implemented using MATLAB (R2025a). The confusion matrix of the CNN test data with seven feature extraction blocks is presented in Figure 7. The evaluation of the classification model, based on a balanced dataset of 5000 samples across 10 classes (500 samples per class), demonstrates exceptional performance. By analyzing the provided confusion matrix visualization, we identified the True Positives (TPs) for each class, which sum to 4845 correctly classified samples. Consequently, the Accuracy is calculated as 4845/5000, resulting in 96.9%. Given the perfectly balanced class distribution, the Macro-Recall is mathematically equivalent to the accuracy at 0.969. These metrics indicate that the model achieves a consistently high level of precision and recall across all categories, with only minimal misclassifications totaling 155 samples.

**Figure 8:**
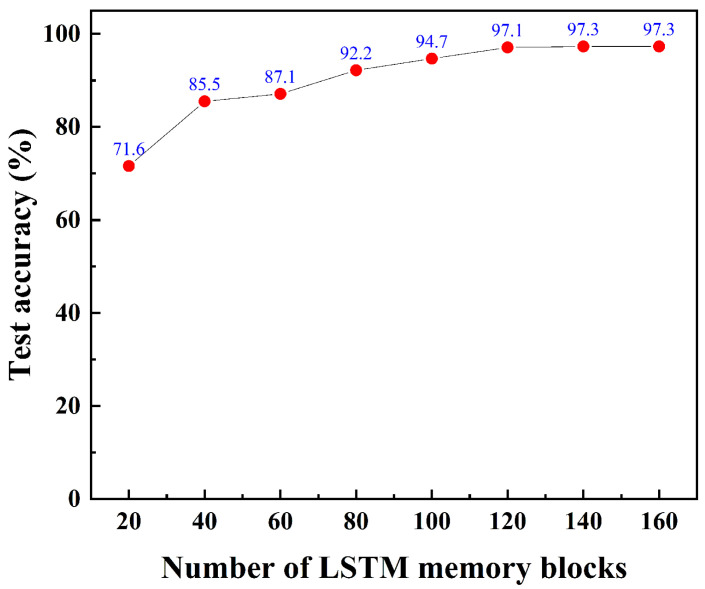
Relationship between test accuracy and number of LSTM blocks. The optimizer with 140 or 160 LSTM memory blocks achieved the highest test accuracy.

**Figure 9:**
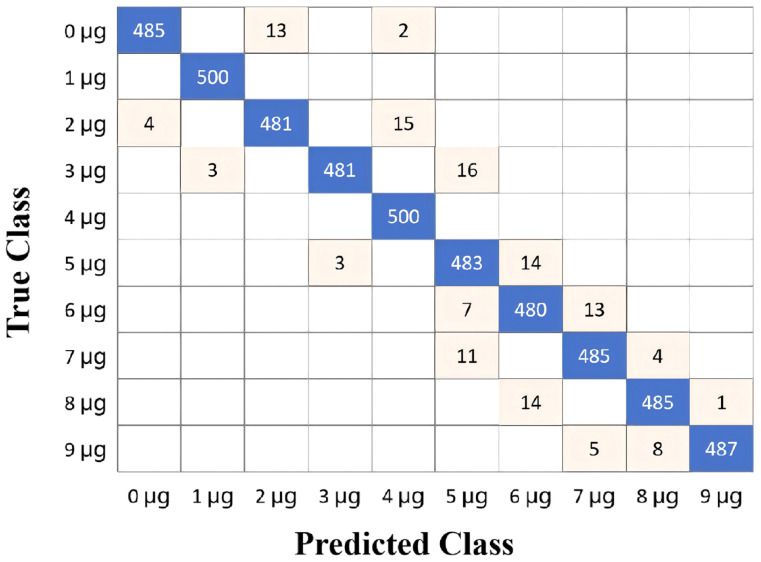
Confusion matrix for test data showing the classification results using the LSTM model with 140 memory blocks. (*n* = 5000 samples; 500 per class: 0 to 9 μg; Overall accuracy: 97.3%).

The relationship between the average test accuracy and the number of LSTM blocks is shown in Figure 8. The test accuracy improved with an increase in the number of memory blocks. Specifically, the test accuracy surpassed 90% when the number of LSTM memory blocks was increased to 80. The highest test accuracy achieved was 97.3% with 140 or 160 memory blocks. All procedures were implemented using MATLAB (R2025a). The confusion matrix of the LSTM test data with 140 memory blocks is presented in Figure 9. Based on the confusion matrix for a balanced dataset of 5000 samples (10 classes × 500 samples each), the model demonstrates excellent performance with an Accuracy of 97.3% (4867 correct predictions out of 5000). Since the class distribution is perfectly balanced, the Macro-Recall is mathematically identical to the Accuracy at 0.973. Assuming a symmetric error distribution where Precision approximates Recall, the Macro-F1 score is also estimated to be approximately 0.973. The analysis reveals that two classes achieved perfect classification (500/500), while the remaining classes showed very high accuracy, with Class 9 μg achieving the highest non-perfect score of 487/500 and Class 2 μg and 3 μg showing the lowest (but still strong) scores of 481/500. These metrics indicate a highly reliable model with consistent performance across all categories.

**Figure 10:**
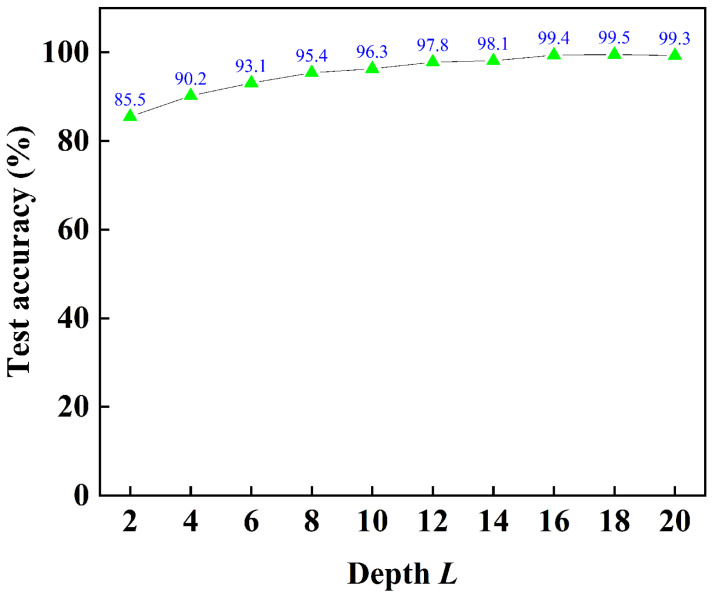
Relationship between test accuracy and the depth *L* of the Transformer model.

**Figure 11:**
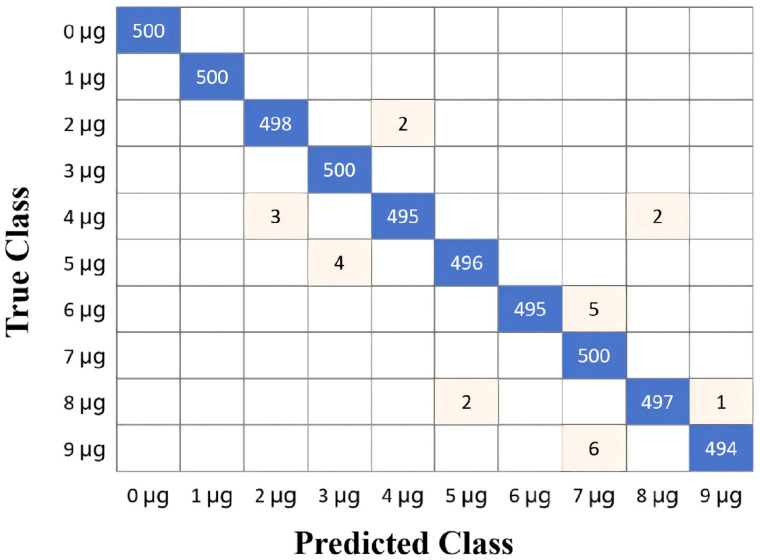
Confusion matrix for test data showing the classification results using the Transformer. (*n* = 5000 samples; 500 per class: 0 to 9 μg; Overall accuracy: 99.5%).

The test accuracy is closely related to the depth *L* of the Transformer model. Increasing the depth *L* usually enhances the model’s representational ability, enabling it to learn deeper and more abstract feature representations. However, increasing the depth also brings some challenges, such as gradient vanishing/explosion, exponential growth in computational cost, and an increased risk of overfitting. The relationship between the test accuracy and the depth *L* of the Transformer model is shown in Figure 10. When *L* is set to 18, the test accuracy attains its peak value of 99.5%. The experimental setup was implemented using the PyTorch (Version 2.6.0) framework. A specific virtual environment was established using Anaconda (Version 25.7.0) to ensure reproducibility and manage package dependencies, primarily relying on the PyTorch, NumPy, and Scikit-learn packages. The code was developed and executed in a PyCharm development environment (Version 2025.1). The confusion matrix of the Transformer test data with the depth *L* = 18 is presented in Figure 11. Based on the confusion matrix for a balanced dataset of 5000 samples (10 classes × 500 samples each), the model achieves exceptional performance with an Accuracy of 99.5% (4975 correct predictions out of 5000). Given the perfectly balanced class distribution, the Macro-Recall is mathematically identical to Accuracy at 0.995. Under the assumption of symmetric error distribution typical in high-performing models, the Macro-F1 score is also estimated to be approximately 0.995. The analysis reveals that four classes were classified perfectly (500/500), while the remaining six classes showed minimal errors, with Class 9 μg having the highest misclassification count (6 errors) and Class 2, 4, 5, 6, and 8 μg each exhibiting only 2–5 mistakes. These results indicate a near-flawless classifier with remarkable consistency across all categories, totaling just 25 misclassifications overall.

**Figure 12:**
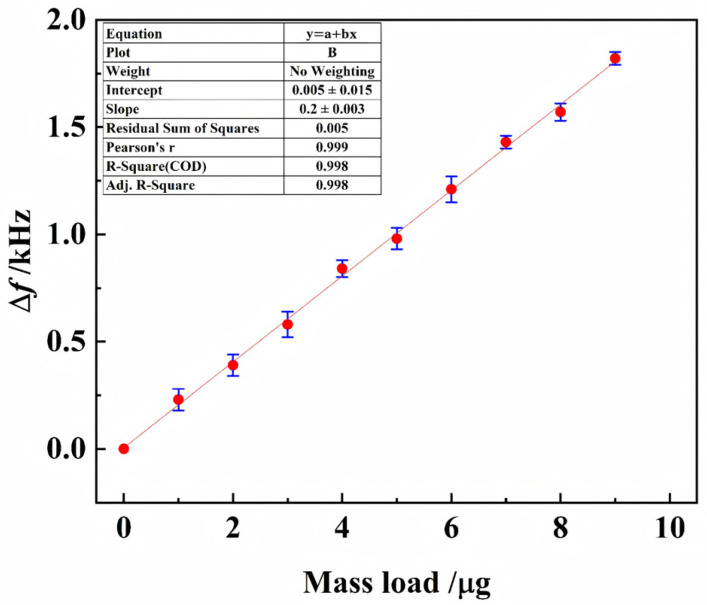
Relationship of frequency shift (Δ*f*) and mass load (Δ*m*).

An impedance analyzer (Agilent 4294A, Agilent Technologies, Santa Clara, CA, USA) was used to measure the impendence spectrum of the PVDF piezoelectric microbalance sensor. The resonant frequency of the PVDF piezoelectric microbalance is approximately 8.5 kHz. The relationship between mass load and frequency change is shown in Figure 12. Experimental data points conform precisely to a linear regression model (dashed line; R^2^ > 0.99), with the magnitude of Δ*f* scaling linearly with Δ*m*.

### 4.2. Discussion of Results

This study introduced a portable PTFM sensing platform that utilizes acoustic signal transduction integrated with three DL architectures, namely CNN, LSTM, and transformers, for precise mass quantification. Experimental validation confirmed the exceptional efficacy of the system in POCT scenarios, with critical insights derived from the comparative algorithmic performance and system-level integration. The Transformer model achieved superior classification accuracy (99.5%) in mass-loading detection tasks, significantly outperforming the CNN (96.9%) and LSTM (97.3%). This performance advantage is attributed to the capacity of the self-attention mechanism to model long-range temporal dependencies. In contrast, CNNs exhibit robustness in local feature extraction but demonstrate inherent limitations in capturing global contextual dynamics. LSTM networks mitigate vanishing gradient issues through gated recurrent units but remain constrained by sequential processing bottlenecks when handling high-dimensional spectro-temporal features.

## 5. Conclusions

### 5.1. Summary of Research Findings

The key innovation of this study lies in the tripartite integration of sensing hardware (PVDF-based PTFM), edge-compatible algorithms, and smartphone-based acoustic acquisition. This decentralized paradigm eliminates the reliance on bulky laboratory instrumentation (e.g., network analyzers) and achieves analytical precision while reducing the cost, size, and operational complexity. Such portability holds transformative potential for resource-constrained settings, including rural clinics, field environmental monitoring, and wearable health diagnostics, effectively bridging the gap between centralized laboratory analysis and rapid on-site screening.

Parametric sensitivity analysis further elucidated architecture-specific optimization thresholds: CNN performance saturated at seven convolutional blocks, LSTM peaked with 140 or 160 memory blocks, and the Transformer attained maximal accuracy at 18 encoder layers. These empirical thresholds reflect critical trade-offs between model capacity, computational overhead, and the information density inherent in acoustic response signatures, providing actionable guidelines for future embedded system deployment.

Unlike previous piezoelectric sensing platforms that focused exclusively on materials optimization or electrical readout improvements, our work introduces a holistic reimagining of the sensing paradigm through two interconnected innovations: (1) repurposing acoustic signals as the primary information carrier rather than a byproduct to be minimized; (2) implementing DL architectures specifically optimized for extracting mass-loading information from complex acoustic signatures. This integrated approach transcends incremental improvements to existing methodologies by fundamentally reconfiguring the relationship between sensing physics, signal transduction, and information extraction—enabling a new portable, high-accuracy micro-mass detection system.

### 5.2. Research Limitations and Future Research Efforts

Nevertheless, this study has several limitations that warrant discussion. First, the validation experiments were conducted under controlled laboratory acoustics, and the robustness of the proposed method against real-world ambient noise (e.g., clinical or outdoor environments) remains to be established. Second, the detection specificity was assessed solely using single-stranded DNA analytes; extension to complex biological matrices such as whole blood or saliva will require multi-analyte calibration and effective interference mitigation strategies. Third, the intrinsic quality factor (*Q*-factor) of the PVDF microbalance sensor imposes a fundamental limitation on the achievable mass resolution. For the PVDF microbalance sensor used in this work (thickness: 28 μm, diameter: 5 mm), the *Q*-factor was measured to be 27.6 at a resonance frequency of 8.5 kHz, corresponding to a half-power bandwidth of 0.308 kHz. Such a relatively low *Q*-factor, typical for polymer-based piezoelectric materials owing to their higher mechanical damping and lower electromechanical coupling compared to ceramic piezoelectrics, broadens the resonance peak and degrades the frequency stability. Consequently, the sensor’s resolution is constrained when attempting to detect minute mass changes at the sub-nanogram level, as frequency shifts become more difficult to resolve against the increased noise floor.

To overcome these limitations, future research will concentrate on three main directions: (i) developing adaptive noise-suppression algorithms combined with differential sensing configurations to mitigate environmental disturbances; (ii) designing multimodal fusion frameworks that integrate complementary transduction principles (e.g., impedance and mass sensing) for improved specificity in heterogeneous samples; and (iii) exploring alternative piezoelectric materials with intrinsically higher *Q*-factors, such as AlN, KNN or PZT. These material choices would directly address the *Q*-factor bottleneck and push the detection limit toward the sub-nanogram regime.

In conclusion, this study establishes a robust and application-oriented foundation for acoustic-driven mass sensing in decentralized diagnostic applications. By synergistically integrating materials science, signal processing, and AI, the proposed platform significantly enhances the potential for accessible, precise, and scalable POCT solutions. As edge computing capabilities continue to advance and new piezoelectric composites emerge, this framework is well positioned to accelerate translational adoption in precision medicine, environmental monitoring, and personalized healthcare.

## Data Availability

The authors confirm that the data supporting the findings of this study are available within the article.
